# Clinical significance and long-term prognosis of ulcerative colitis patients with appendiceal orifice inflammation

**DOI:** 10.1186/s12876-022-02627-w

**Published:** 2022-12-21

**Authors:** Kyong Wook Kuk, Ji Yeon Gwon, Jae Seung Soh, Hyun Lim, Ho Suk Kang, Sung-Hoon Moon, Abebe Muraga, Ji Won Yoo

**Affiliations:** 1 Department of Internal Medicine, Hallym University Sacred Heart Hospital, University of Hallym College of Medicine, 22 Gwanpyeong-ro 170-gil, Dongan-gu, Anyang, 14068 Republic of Korea; 2 Department of Internal Medicine, Kirk Kerkorian School of Medicine, University of Nevada Las Vegas, Las Vegas, NV USA

**Keywords:** Appendiceal orifice inflammation, Ulcerative colitis, Prognosis, Colonoscopy

## Abstract

**Background:**

Appendiceal orifice inflammation (AOI) or peri-appendiceal red patch is a skip lesion with segments of continuous colitis from the rectum. Frequently observed in ulcerative colitis (UC) patients, this lesion might be associated with proximal extension in some studies. However, the clinical significance of this lesion and long-term outcomes including therapy remain unclear. Thus, the aim of this study was to evaluate the clinical implication of AOI during long-term periods in patients with UC.

**Methods:**

We retrospectively reviewed 376 patients with UC who performed complete colonoscopic examinations between April 2000 and December 2020. We compared clinical characteristics and outcomes of patients manifesting AOI with those who did not show AOI during a mean follow-up period of 66.1 months. Long-term outcomes included maximal extent of colitis, proximal extension, therapeutic medical histories, UC-related hospitalization, and relapse.

**Results:**

Ninety-eight (26.1%) patients showed AOI without evidence of inflammation in the right colon. Mild disease activity at the diagnosis of UC was more included in patients with AOI than in those without AOI. Other baseline characteristics including disease extent, smoking history, external intestinal manifestation, and terminal ileal ulceration were not significantly different between the two groups. During follow-up periods, patients with and without AOI showed no significant difference in proximal extension, Mayo endoscopic score at the last endoscopic examination, UC-related hospitalization, or relapse. Of medication history, patients with AOI were less included in the group treated with high-dose aminosalicylates than those without AOI. However, therapeutic histories of steroids, immunosuppressive agents, and biologics were not significantly different. Of 62 patients with AOI who underwent follow-up colonoscopy, 36 (58.1%) showed resolution of AOI. Clinical outcomes of the resolution group were not different than those of the non-resolution group. Biopsy results of 77 patients with AOI showed chronic active or erosive colitis.

**Conclusions:**

Long-term outcomes of UC patients with AOI were not different from those without AOI. Outcomes of resolution and non-resolution groups of AOI patients were not different either. Thus, AOI might have no prognostic implication in distal UC patients.

## Introduction

Appendiceal orifice inflammation (AOI) is referred to as peri-appendiceal red patches presented on mucosal erythema, granularity, erosion, and friability by colonoscopy [1]. Since ulcerative colitis (UC) is considered a continuous inflammation from the rectum without any skipped area, AOI could be a compounding factor for the diagnosis of UC. In addition, physicians have concerns about whether to add oral 5-aminosalicylic acid (5-ASA) to suppository 5-ASA for the treatment of patients with UC proctitis accompanying AOI. In addition, AOI might be considered as a remnant inflammation that occurs after medical therapy.

AOI is more frequently observed in UC than in other colitis. It could lead to an effective diagnosis of UC when it is combined with proctitis [2]. The reported prevalence of AOI ranges from 7.9–75.0% in UC patients observed endoscopically [3]. Such large difference in the prevalence of AOI might be due to differences in the number of enrolled patients and the presence or absence of extensive colitis. The clinical course of AOI shows different results among studies. One study has reported that AOI is correlated with subsequent proximal extension of mucosal inflammation in UC patients [4]. Controversially, another study has reported that involvement of the appendiceal orifice might be indicative of responding well to pharmacotherapy [5]. Other studies have also concluded that AOI does not have any prognostic implication such as disease severity, relapse, or medical therapy [6, 7]. The clinical significance of AOI in patients with UC needs to be fully evaluated.

Thus, the aim of this study was to investigate the frequency of AOI in UC patients with distal inflammatory involvement and determine the clinical significance and long-term prognosis of UC patients with AOI compared with those without AOI.

## Methods

### Study patients

Medical records of patients diagnosed with UC at the Hallym University Sacred Heart Hospital in Anyang, Korea between April 2000 and December 2020 were analyzed retrospectively. The clinical significance and natural course of discrete terminal ileal ulcers in UC patients using these data have been published [8]. The previous study enrolled 397 patients diagnosed with UC who underwent a colonoscopic examination with successful terminal ileal intubation. Twenty-one patients having inflammatory mucosal change including scar or pseudopolyp in ascending colon after reinvestigating colonoscopic findings were excluded. The following characteristics of the remaining 376 patients were analyzed: sex, age of UC diagnosis, date of the last follow-up, smoking history, family history, extraintestinal manifestation, disease extent and activity, history of treatment with 5-ASA, corticosteroids, azathioprine and/or 6-mercaptopurine (6-MP), anti-tumor necrotizing factor (TNF) agents, history of colectomy or UC-related hospitalization, and relapse. All patients enrolled in this study were treated with oral and/or suppository 5-ASA. We defined patients treated with more than 3 g of oral 5-ASA per day as those with high-dose 5-ASA. The disease extent was determined based on colonoscopic findings: only involved in the rectum as proctitis, disease up to the splenic flexure as left-sided, and disease beyond the splenic flexure as extensive. Disease activity was categorized as inactive (score of 0–2), mild (score of 3–5), moderate (score of 6–10), or severe (score of 11–12) based on the Mayo score [9].

### Clinical and colonoscopic outcomes

We evaluated the following outcomes during follow-up periods. Maximal disease extent was defined as maximum disease extent according to the above disease extent. Proximal extension was defined as proximal progression of colonic inflammation (e.g., proctitis to left-sided or extensive colitis, or left-sided to extensive colitis). UC-related hospitalization was defined as having histories of admission at our hospital for UC aggravation or adverse events of medication. Relapse was defined by the use of corticosteroids, azathioprine, 6-MP, or anti-TNF agents followed by clinical or endoscopic aggravation of mucosal inflammation. Colonoscopic examinations were performed by staff and fellows at the department of gastroenterology of Hallym University Sacred Heart Hospital. We evaluated whether AOI remained or resolved through follow-up colonoscopies. Terminal ileal ulcers were defined as definite mucosal breaks on terminal ileum according to our previous study [8]. Histology was evaluated by gastrointestinal pathologists who were skilled at histological analysis of inflammatory bowel disease (IBD). The study protocol was approved by the Institutional Review Board of Hallym University Sacred Heart Hospital, Anyang, Korea (No. 2022-03-006-001). It was conducted in accordance with relevant guidelines and regulations.

### Statistical analyses

Clinical manifestations and outcome parameters were compared between patients with AOI and those without AOI. Statistical significance was considered at *P* value < 0.05. Continuous variables were compared by Mann-Whitney *U* test and categorical variables were compared with Chi-square test or two-tailed Fisher’s exact test. History of treatment with azathioprine/6-MP and anti-TNF agents, UC-related hospitalization, and relapse were calculated using the Kaplan–Meier method, and subgroups were compared by the log rank test. The Statistical Package for the Social Sciences (SPSS) version 25.0 (SPSS Inc., Chicago, IL, USA) was used for all statistical analyses.

## Results

### Comparison of baseline clinical features between patients with and without AOI

**Fig. 1:**
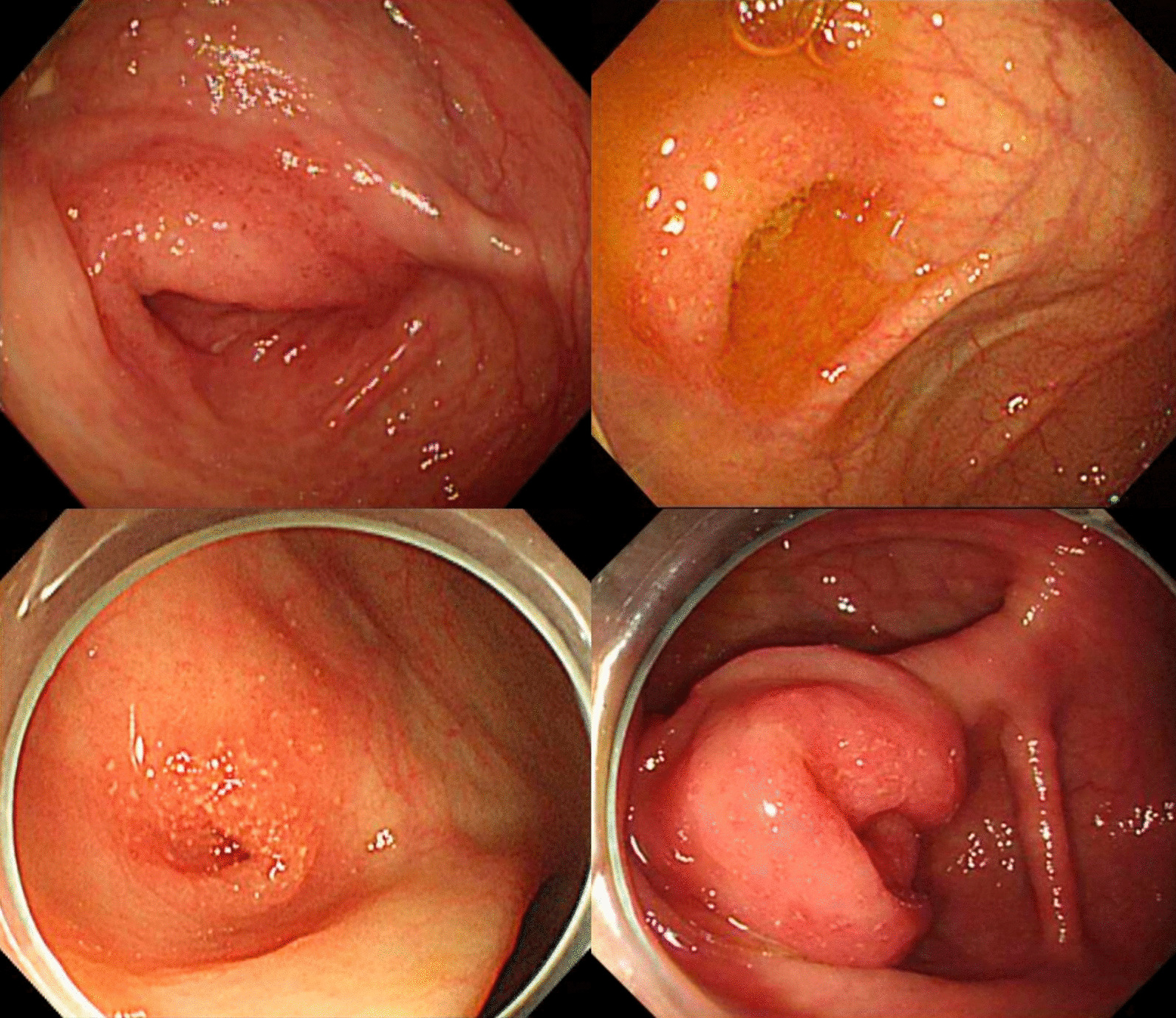
Colonoscopic images of appendiceal orifice inflammation in patients with ulcerative colitis

Of the 376 UC patients, 98 (26.1%) had AOI in colonoscopic examination. Figure 1 shows images of AOI in UC patients of our study. We compared these 98 patients with AOI (AOI positive) with the 278 patients without AOI (AOI negative). Mild to moderate disease activity was more in the AOI positive group than in the AOI negative group (69.4% *vs.* 54.3%, *P* = 0.012) (Table 1). However, there was no significant difference in age, sex, smoking history, family history, the prevalence of terminal ileal ulcer, or the occurrence of extraintestinal manifestations (e.g., arthritis, stomatitis, ankylosing spondylitis, psoriasis, uveitis, erythema nodosum, primary sclerosing cholangitis, and epididymitis) between the two groups. Disease extent at the diagnosis of UC showed no significant difference between the two groups either (*P* = 0.288). Among 10 patients who discontinued 5-ASA due to adverse events (8 for pruritus and/or skin lesions, 1 for hepatitis, 1 for pericarditis and pancreatitis), 2 patients were in AOI positive group, and the resting 8 patients were in AOI negative group (*P* = 1.000).

**Table 1:** Baseline clinical manifestations at the diagnosis of UC between patients with and without AOI

Variables	AOI positive (n = 98)	AOI negative (n = 278)	*p* value
Age, years, median (range)	44 (13–84)	39 (11–80)	0.108
Male sex, no. (%)	66 (67.3)	167 (60.1)	0.227
*Smoking history, no. (%)*			0.549
Current/previous, no. (%)	42 (42.9)	108 (38.8)	
Never, no. (%)	56 (57.1)	170 (61.2)	
Family history, no. (%)	1 (1.0)	5 (1.8)	1.000
Extraintestinal manifestation, no. (%)	12 (12.2)	33 (11.9)	1.000
Terminal ileal ulcer, no. (%)	9 (9.2)	30 (10.8)	0.847
*Disease activity*			**0.012**
Mild/inactive, no (%)	68 (69.4)	151 (54.3)	
Moderate/severe, no. (%)	30 (30.6)	127 (45.7)	
*Disease extent*			0.288
Proctitis, no. (%)	60 (61.2)	150 (54.0)	
Left-sided colitis, no. (%)	22 (22.4)	76 (27.3)	
Extensive colitis, no. (%)	16 (16.3)	52 (18.7)	

*P* value < 0.05 was regarded as statistically significant and is designated in bold

*AOI* appendiceal orifice inflammation, *SD* standard deviation, *UC* ulcerative colitis

### Comparison of clinical outcomes between patients with and without AOI during follow-up periods

**Fig. 2:**
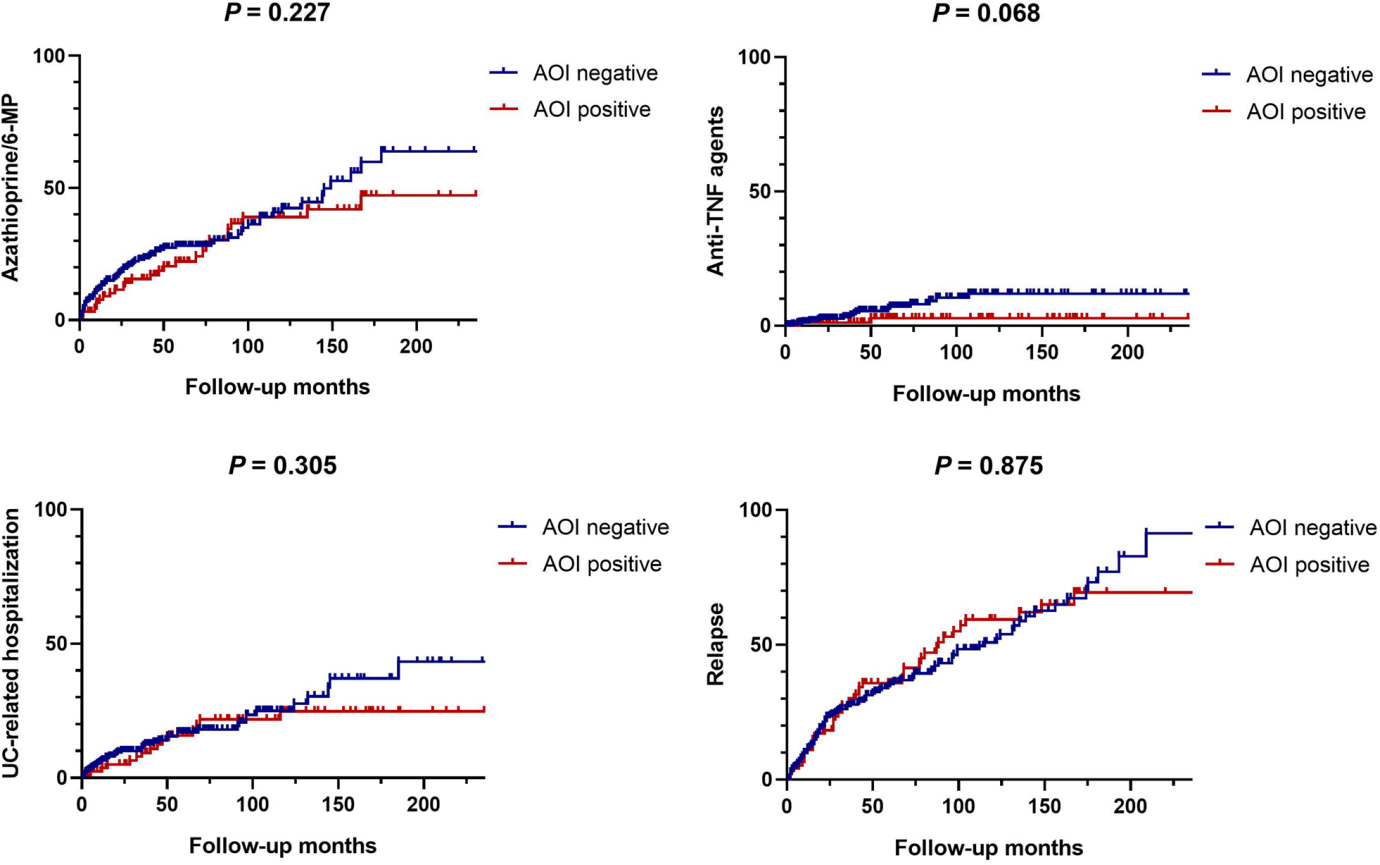
Comparison between the AOI positive group and AOI negative group in history of treatment with azathioprine/6-MP and anti-TNF agents, UC-related hospitalization, and relapse using Kaplan–Meier methods

The median follow-up duration was 56.5 months for the AOI positive group and 52.0 months for the AOI negative group, showing no significant difference between the two groups (*P* = 0.285). Maximal extent, proximal extension, and Mayo endoscopic score at the last endoscopy during follow-up periods were not significantly different between the two groups either (Table 2). Twenty-four (24.5%) and 50 (18.0%) patients showed proximal extension in the AOI positive group and the AOI negative group, showing no significant difference between the two groups (*P* = 0.184). The administration of a high dose 5-ASA was lower in the AOI positive group than in the AOI negative group (13.3% vs. 24.8%, *P* = 0.016). However, use of medications including corticosteroids, azathioprine/6-MP, and anti-TNF agents was not significantly different between the two groups. A total proctocolectomy was performed for two patients in the AOI negative group. UC-related hospitalization and relapse during follow-up periods were not significantly different between the two groups (*P* = 0.894 and 0.338, respectively). In the analysis of the clinical outcomes relative to time, there were no significant differences between both groups in the history of treatment with azathioprine/6-MP and anti-TNF agents, UC-related hospitalization, and relapse (Fig. 2).

**Table 2:** Clinical outcomes during follow-up periods between patients with and without AOI

Variables	AOI positive (n = 98)	AOI negative (n = 278)	*p* value
*Maximal extent, no. (%)*			0.699
Proctitis, no. (%)	39 (39.8)	115 (41.4)	
Left-sided colitis, no. (%)	29 (29.6)	84 (30.2)	
Extensive colitis, no. (%)	30 (30.6)	79 (28.4)	
Proximal extension, no. (%)	24 (24.5)	50 (18.0)	0.184
*Mayo endoscopic score at the last endoscopy*			0.516
0–1, no. (%)	73 (74.5)	196 (70.5)	
2–3, no. (%)	25 (25.5)	82 (29.5)	
*Histories of medications*			
High dose 5-ASA, no (%)	13 (13.3)	69 (24.8)	**0.016**
Corticosteroid, no. (%)	53 (54.1)	150 (54.0)	1.000
Azathioprine/6-MP, no. (%)	27 (27.6)	86 (30.9)	0.609
Anti-TNF agents, no. (%)	2 (2.0)	17 (6.1)	0.177
Colectomy, no. (%)	0 (0.0)	2 (0.7)	1.000
UC-related hospitalization, no. (%)	25 (25.5)	75 (27.0)	0.894
Relapse during follow-up periods, no. (%)	44 (44.9)	108 (38.8)	0.338
Follow-up duration, months, median (range)	56.5 (1-186)	52.0 (1-245)	0.285

*P* value < 0.05 was regarded as statistically significant and is designated in bold

*AOI* appendiceal orifice inflammation, * 5-ASA* 5-aminosalicylic acid, * MP* mercaptopurine, *TNF* tumor necrosis factor, *UC* ulcerative colitis, *SD* standard deviation

### Comparison of clinical outcomes according to AOI resolution

Of the 98 patients with AOI, 36 patients did not undergo follow-up colonoscopy. Of the remaining 62 patients, 36 (58.1%) showed resolution of AOI during the follow-up colonoscopy. The median follow-up duration of these patients was 83 months (range, 1–185 months) and the median number of follow-up colonoscopies was 4 times (range, 2–7 times). The median follow-up duration of 26 patients with continuing AOI was 84 months (range, 12–168 months) and the median number of follow-up colonoscopies was 4 times (range, 2–6 times). We compared clinical features between the AOI resolution group and the AOI continuing group. Baseline characteristics including disease activity and extent were not significantly different. Clinical outcomes during follow-up periods including maximal extent, proximal extension, Mayo endoscopic score at the last endoscopy, medication histories, UC-related hospitalization, and relapse were not significantly different between the two groups either (Table 3).

**Table 3:** Clinical outcomes during follow-up periods according to resolution of AOI

Variables	Resolution (n = 36)	No resolution (n = 26)	*p* value
*Maximal extent, no. (%)*			0.656
Proctitis, no. (%)	12 (33.3)	9 (34.6)	
Left-sided colitis, no. (%)	14 (38.9)	7 (26.9)	
Extensive colitis, no. (%)	10 (27.8)	10 (38.5)	
Proximal extension, no. (%)	9 (25.0)	8 (30.8)	0.774
*Mayo endoscopic score at the last endoscopy*			0.177
0–1, no. (%)	32 (88.9)	19 (73.1)	
2–3, no. (%)	4 (10.8)	7 (26.9)	
*Histories of medications*			
High dose 5-ASA, no (%)	3 (8.3)	4 (15.4)	0.439
Corticosteroid, no. (%)	21 (58.3)	16 (61.5)	1.000
Azathioprine/6-MP, no. (%)	10 (27.8)	10 (38.5)	0.419
Anti-TNF agents, no. (%)	0 (0.0)	0 (0.0)	
Colectomy, no. (%)	0 (0.0)	0 (0.0)	
UC-related hospitalization, no. (%)	7 (19.4)	9 (34.6)	0.242
Relapse during follow-up periods, no. (%)	18 (50.0)	13 (50.0)	1.000
Follow-up duration, months, median (range)	83 (1-185)	84 (12–168)	0.272

*AOI* appendiceal orifice inflammation, *5-ASA* 5-aminosalicylic acid, *MP* mercaptopurine, *TNF* tumor necrosis factor, *UC* ulcerative colitis, *SD* standard deviation

### Histologic features of AOI

Seventy-seven (78.6%) patients had undergone biopsies of AOI lesions. All biopsies were pathologically diagnosed with chronic active or erosive colitis with or without lymphoid hyperplasia. Twenty-two (28.6%) patients were additionally diagnosed with cryptitis, crypt distortion, and crypt abscess as histologic findings indicating active UC.

## Discussion

In this study, we found that long-term outcomes including proximal extension, histories of medications, UC-related hospitalization, and relapse of UC patients with AOI were not significantly different from those without AOI. Long-term outcomes were not significantly different between the group with AOI resolved and the group with AOI remained. Clinical implications of AOI in UC patients with distally involved colitis did not show a significance in the natural course.

Patchy inflammation endoscopically observed on appendiceal orifice in distal UC patients was prospectively evaluated in 1997 [10]. Since then, several studies have reported its prevalence, clinical significance, and natural outcomes. Because the general inflammatory feature of UC proximally progresses from the rectum with continuity, skip patchiness on the cecum is considered a lesion that occurs after medical treatment [11]. However, one study has suggested that AOI is not the result of medical therapy by excluding UC patients who show prior involvement in ascending colon and comparing the prevalence of AOI in newly diagnosed patients with that in preexisting diagnosed patients [1]. After that, AOI has been recognized as a patchy inflammation on the cecum separate from segments of continuous involvement from the rectum.

The prevalence of AOI in UC varies between studies, ranging from 7.9–75% because of different diagnostic criteria used. A few studies have evaluated the prevalence of AOI in examinations performed regardless of treatment history or disease duration [5, 10, 12] while other studies have assessed the prevalence only in newly diagnosed with UC [1, 7]. Studies showing a high prevalence of AOI enrolled UC patients with pancolitis or evaluated with a small number of patients [10, 13]. In our study, the prevalence of AOI was 26.1%. We included many UC patients who underwent total colonoscopy over a long-term period. The prevalence was not different from previous studies having similar inclusion criteria [1, 14]. Although we enrolled 68 (18.1%) patients with pancolitis in this study, we investigated all colonoscopic examinations of patients to evaluate inflammation in the cecum and ascending colon.

The significance of AOI also varies among studies reporting different prognoses, disease severity, and risk of proximal extension. Strisciuglio et al. have shown that periappendiceal inflammation is associated with a major extent of UC inflammation and that pediatric UC patients with AOI have a higher grade of inflammation at the ascending colon than those without AOI [14]. Anzai et al. [4] reported that nine patients with UC proctitis and AOI showed proximal extension in all patients during follow-up duration, while 17 (44.7%) of 38 proctitis patients without AOI showed proximal extension. Their study suggested that AOI in UC patients with proctitis was correlated with subsequent proximal extension of mucosal inflammation. However, their study had a limitation because of a small number of patients. In our study, there was no significant difference in the prevalence of proximal extension between 61 patients of the AOI positive group and 150 patients of AOI negative group with UC proctitis (34.4% vs. 23.3%, *P* = 0.122). In addition, maximal extent, histories of medication, UC-related hospitalization, and relapse were not significantly different between the two groups diagnosed with proctitis at the diagnosis of UC. Our study included larger number of patients with longer follow-up periods than previous studies.

Our study correlates with other studies in assessing the clinical course. Byeon et al. have reported that AOI has no prognostic implications in terms of remission, relapse, or proximal extension in 94 patients with distal UC [7]. Naves et al. [6] have shown that UC patients with AOI and UC controls show no significant difference in proximal spread, history of using systemic steroids, requirements of rescue therapies, or colectomy. In a meta-analysis published including the above studies, combining with AOI did not affect the courses of UC, the severity of the disease, or the prevalence of surgical treatment [15].

A randomized, double-blind, controlled trial (ASCEND II) showed that patients treated with a high-dose of 5-ASA (4.8 g/dL) achieved significantly more overall improvement after induction therapy of moderately active UC compared with those with standard-dose 5-ASA (2.4 g/dL) [16]. In a meta-analysis based on 12 trials including 2492 patients with mild to moderate UC, high-dose 5-ASA (> 3 g/dL) had a trend to be better than standard-dose 5-ASA (2–3 g/dL) for inducing clinical remission, although the difference in benefit did not reach statistical significance (risk ratio: 0.94 [0.88–1.01]) [17]. In the present study, all patients had medication histories of oral and/or suppository 5-ASA. High-dose 5-ASA might be useful for clinical remission and maintenance in patients with AOI. However, high-dose use of 5-ASA was lower in the AOI positive group than in the AOI negative group. AOI positivity was not a consideration for the decision of 5-ASA dosage in this study. Among patients treated with high-dose 5-ASA, 42.9% showed resolution of AOI during follow-up periods, which was lower than the resolution rate of 60.0% in patients treated with standard or low-dose (< 2 g/dL) 5-ASA.

Histologic evaluation of AOI needs to be performed at the first detection. Iwamuro *et al.* reported a case that biopsy results in the cecum could discriminate AOI from mucosa-associated lymphoid tissue (MALT) lymphoma in UC [18]. In addition, histologic assessment of endoscopically abnormal lesions could distinguish inflammatory lesions from premalignant lesions such as adenomatous lesions and lymphoma. Histologic evaluation could also help assess the grade of inflammation [5, 14]. Our study showed that biopsy results of all patients had chronic active or erosive colitis with or without lymphoid hyperplasia, which were not different from biopsy specimens of another colonic part involved in UC.

The current study was conducted in a retrospective and non-randomized manner at a single center. Biases might have occurred due to unrecognized or unmeasured factors. In addition, patients with extensive colitis at the diagnosis of UC were included, although mucosal inflammation did not reach the ascending colon. The possibility of patchy improvement of inflammation secondary to medical therapy could not be ruled out. Our study had included more patients with mild or inactive disease activity in the AOI positive group. It might lead to less use of high-dose 5-ASA or anti-TNF agents in the AOI positive group than in the AOI negative group. Patients who performed colectomy were also not included in the AOI positive group. Lastly, the history of taking aspirin or non-steroidal anti-inflammatory drug (NSAIDs) and information on bowel preparation were not evaluated. NSAID or preparation agent could contribute to inflammation of the right-sided colon.

In conclusion, patients with AOI at the diagnosis of UC did not show any differences in long-term outcomes such as progression of disease extent, hospitalization, disease relapse, or medication histories compared with patients without AOI. Lesions were resolved in more than half of patients with AOI during follow-up periods. A further study enrolling a large number of patients is needed to support results of this study.

## Data Availability

The datasets used and/or analyzed during the current study are available from the corresponding author upon reasonable request.
